# Cthrc1 Is a Positive Regulator of Osteoblastic Bone Formation

**DOI:** 10.1371/journal.pone.0003174

**Published:** 2008-09-09

**Authors:** Hiroaki Kimura, Kin Ming Kwan, Zhaoping Zhang, Jian Min Deng, Bryant G. Darnay, Richard R. Behringer, Takashi Nakamura, Benoit de Crombrugghe, Haruhiko Akiyama

**Affiliations:** 1 Department of Orthopaedics, Kyoto University, Kyoto, Japan; 2 Department of Molecular Genetics, The University of Texas M. D. Anderson Cancer Center, Houston, Texas, United States of America; 3 Department of Experimental Therapeutics, The University of Texas M. D. Anderson Cancer Center, Houston, Texas, United States of America; 4 Department of Biology, The Chinese University of Hong Kong, Shatin, Hong Kong SAR, China; Fred Hutchinson Cancer Research Center, United States of America

## Abstract

**Background:**

Bone mass is maintained by continuous remodeling through repeated cycles of bone resorption by osteoclasts and bone formation by osteoblasts. This remodeling process is regulated by many systemic and local factors.

**Methodology/Principal Findings:**

We identified collagen triple helix repeat containing-1 (Cthrc1) as a downstream target of bone morphogenetic protein-2 (BMP2) in osteochondroprogenitor-like cells by PCR-based suppression subtractive hybridization followed by differential hybridization, and found that *Cthrc1* was expressed in bone tissues *in vivo*. To investigate the role of Cthrc1 in bone, we generated *Cthrc1*-null mice and transgenic mice which overexpress *Cthrc1* in osteoblasts (*Cthrc1* transgenic mice). Microcomputed tomography (micro-CT) and bone histomorphometry analyses showed that *Cthrc1*-null mice displayed low bone mass as a result of decreased osteoblastic bone formation, whereas *Cthrc1* transgenic mice displayed high bone mass by increase in osteoblastic bone formation. Osteoblast number was decreased in *Cthrc1*-null mice, and increased in *Cthrc1* transgenic mice, respectively, while osteoclast number had no change in both mutant mice. *In vitro*, colony-forming unit (CFU) assays in bone marrow cells harvested from *Cthrc1*-null mice or *Cthrc1* transgenic mice revealed that Cthrc1 stimulated differentiation and mineralization of osteoprogenitor cells. Expression levels of osteoblast specific genes, *ALP*, *Col1a1*, and *Osteocalcin*, in primary osteoblasts were decreased in *Cthrc1*-null mice and increased in *Cthrc1* transgenic mice, respectively. Furthermore, BrdU incorporation assays showed that Cthrc1 accelerated osteoblast proliferation *in vitro* and *in vivo*. In addition, overexpression of Cthrc1 in the transgenic mice attenuated ovariectomy-induced bone loss.

**Conclusions/Significance:**

Our results indicate that Cthrc1 increases bone mass as a positive regulator of osteoblastic bone formation and offers an anabolic approach for the treatment of osteoporosis.

## Introduction

Bone modeling is initiated by osteoblastic differentiation of mesenchymal cells into preosteoblasts. Preosteoblasts then differentiate into functional osteoblasts producing bone matrix proteins, including type I collagen, osteopontin, and osteocalcin to form bone tissues. Bone is constantly remodeled through bone formation by functional osteoblasts and bone resorption by osteoclasts which arise from monocytic cells, and bone mass is maintained by their precise balance [1]–[3]. Recently, significant advances have been made in our understandings of the factors that regulate the balance between osteoblastic bone formation and osteoclastic bone resorption, but the mechanisms have not been fully elucidated.

Bone morphogenetic proteins (BMPs) are potent osteogenic agents that induce differentiation of mesenchymal cells toward an osteoblastic lineage and stimulate the differentiation and functions of osteoblasts during a process of bone modeling [4]–[6]. Postnatally, BMP signaling regulates osteoblastic bone formation and osteoclastic bone resorption in bone remodeling [7]. Recent studies show that osteoblast-specific inhibition of BMP signals in mice affects postnatal bone mass [8]–[12]. Osteoblast-specific *Bmpr1a* deficient mice show reduced bone volume and bone formation rates with normal osteoblast number due to impaired osteoblast function at 3 months old and increased bone volume probably due to reduced osteoclastic bone resorption at 10 months old [11]. Transgenic mice that osteoblast-specifically overexpress *noggin*, the BMP inhibitor, display reduced bone mineral densities and bone formation rates due to impaired osteoblast function [8], [9]. Mice carrying a targeted deletion of *Tob*, which is a member of a new antiproliferative protein family and represses BMP-induced Smad-dependent transcription in osteoblasts, have increased bone mass due to increased osteoblast numbers and acceleration of the bone formation rates [12]. Thus, BMPs are key molecules that control bone modeling and bone remodeling.

To identify the molecules that regulate bone modeling and/or bone remodeling as a downstream target of BMP signaling, we compared the mRNAs expressed in BMP2-untreated and BMP2-treated ATDC5 osteochondroprogenitor-like cells by PCR-based suppression subtractive hybridization followed by differential hybridization, and identified a cDNA upregulated by BMP2, that encoded Collagen triple helix repeat containing protein 1 (Cthrc1). Cthrc1 is a 30-kD glycosylated secreted protein containing a short collagen-like motif with 12 Gly-X-Y repeats similar to the collagen domains present in the C1q/tumor necrosis factor α related proteins [13]. *Cthrc1* is transiently expressed in the arterial wall in response to injury, suggesting that Cthrc1 is involved in vascular remodeling by limiting collagen matrix deposition and promoting cell migration [13]. A recent study shows that neointimal lesion formation and adventitial collagen deposition in response to carotid artery ligation are reduced in transgenic mice overexpressing *Cthrc1* under the control of cytomegalovirus promoter [14]. According to expression analysis of *Cthrc1*, abundant expression of *Cthrc1* is observed in developing skeleton during embryogenesis and in the bone matrix and periosteum in adult mice [15].

In this study, we generated *Cthrc1*-null mice and transgenic mice that overexpress *Cthrc1* under the control of *Col1a1* osteoblast-specific promoter. *Cthrc1*-null mice developed a low-bone-mass-phenotype as a result of reduced bone formation, while the transgenic mice exhibited a high-bone-mass-phenotype caused by enhanced bone formation. *In vitro* analyses showed that Cthrc1 stimulated osteoblast proliferation and differentiation. Thus, our results indicate that Cthrc1 is a positive regulator of osteoblastic bone formation.

## Results

### *Cthrc1* is expressed in bone

To identify potential osteoblast-specific proteins as downstreams of BMPs, we performed two-step screening consisting of PCR-based suppression subtractive hybridization followed by differential hybridization using the poly(A)^+^ RNA extracted from BMP2-untreated and BMP2-treated ATDC5 cells, and identified a cDNA encoding Cthrc1(Figure S1A). *In vitro*, *Cthrc1* was expressed in MC3T3-E1 osteoblastic cells and differentiated ATDC5 cells, but not in C3H10T1/2 fibroblastic cells and C2C12 myoblastic cells (Figure S1B). We next assessed the expression of *Cthrc1* in various adult mouse tissues by northern blot analysis, and *Cthrc1* was expressed in bone but not in the other adult mouse tissues examined (Figure S1C). During the skeletogenesis in the limb buds of mouse embryos, *Cthrc1* was expressed in the mesenchymal condensation in E13.5 and in the bone tissues in E16.5 (Figure S1D). In addition, the expression levels of *Cthrc1* increased during mouse embryogenesis (Figure S1E). These results suggest that Cthrc1 plays an important role in bone modeling or remodeling.

### Low bone mass and decreased bone formation in *Cthrc1*-null mice

**Figure 1:**
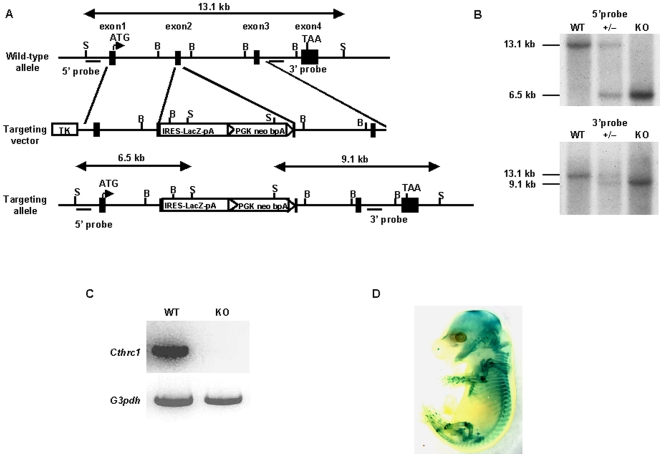
Generation of *Cthrc1*-null mice. (A) Structure of the genomic *Cthrc1* locus, targeting vector, and targeting allele. Exons are depicted as closed boxes, and intronic sequences are shown as solid lines. IRES-LacZ-pA-loxP-flanked PGK-neo-bpA cassettes are depicted as open boxes. S, SacI; B, BamHI. (B) Southern blot analysis of fetal genomic DNA. Genomic DNA isolated from the skin was digested with SacI and then hybridized with the 5′ or 3′ probe. The wild-type and the mutant allele were detected with the 5′ probe as 13.1-kb and 6.5-kb fragments and with the 3′ probe as 13.1-kb and 9.1-kb fragments, respectively. (C) RT-PCR analysis of *Cthrc1* transcript in E16.5 wild-type and *Cthrc1*-null littermates. (D) Whole-mount X-gal staining of E16.5 heterozygous *Cthrc1* embryo. WT: wild-type mice; KO: *Cthrc1*-null mice; +/−: *Cthrc1* heterozygous mice.

**Figure 2:**
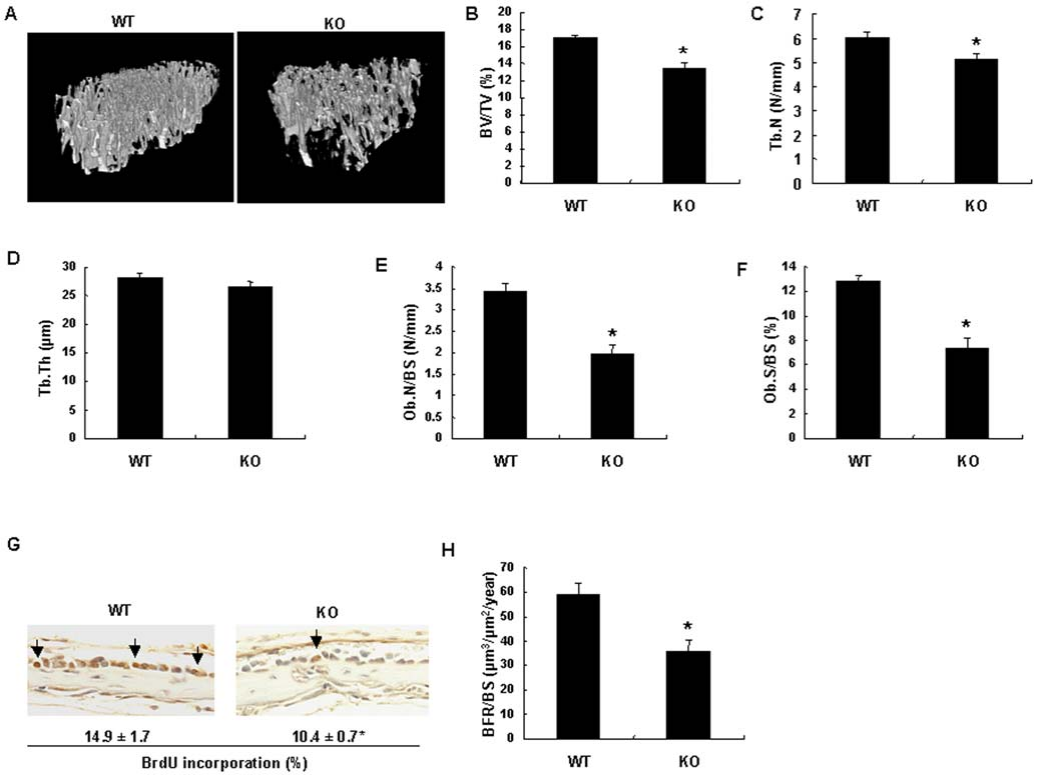
Bone phenotypes of *Cthrc1*-null mice. (A–D) Micro-CT analyses of tibiae in 2-month-old *Cthrc1*-null and wild-type mice (n = 6). Bone volume/total volume (BV/TV), trabecular number (Tb.N), trabecular thickness (Tb.Th) are shown. (E and F) Bone histomorphometric analyses of vertebrae in 2-month-old *Cthrc1*-null and wild-type mice (n = 6). Osteoblast number/bone surface (Ob.N/BS) and osteoblast surface/bone surface (Ob.S/BS) are shown. (G) Analyses of osteoblast proliferation in calvaria of 1-week-old *Cthrc1*-null and wild-type mice by BrdU incorporation assays. Arrows indicate BrdU positive osteoblasts. (H) Calcein double-labeling of vertebrae in 2-month-old *Cthrc1*-null and wild-type mice. BFR : Bone formation rate. WT: wild-type mice; KO: *Cthrc1*-null mice. Data are shown as the mean±SEM (^*^ *p*<0.05).

To investigate the physiological role of Cthrc1 in bone, we inactivated the *Cthrc1* gene in mouse embryonic stem cells by homologous recombination. In the target strategy, an *IRES-lacZ-pA-loxP*-flanked neomycin resistance expression cassette was introduced into exon 2 which codes a short Gly-X-Y collagen triple helix repeat domain (Figure 1 A and B). As shown in Figure 1C, Cthrc1 RNA was absent in E16.5 *Cthrc1*-null embryo, indicating that the mutation was a null mutation. Homozygous *Cthrc1* mutant mice were born at the expected Mendelian ratio and grew normally in comparison with their wild-type littermates (data not shown). X-gal staining of heterozygous *Cthrc1* mutant embryos revealed the expression of *Cthrc1* in bone-forming tissues in E12.5 embryos and in bones and periarticular cartilages in E16.5 embryos, which corresponded to the results of *in situ* hybridization (Figure 1D and Figure S2A). Skeletal preparations showed no obvious skeletal phenotypes in *Cthrc1*-null newborn mice (Figure S2B). *In situ* hybridization analyses showed comparable expression of osteoblast marker genes, *Runx2* and *Col1a1*, and chondrocyte marker genes, *Col2a1* and *Col10a1* between E16.5 *Cthrc1*-null embryos and their wild-type littermates (Figure S3A), suggesting that Cthrc1 has no apparent effect on skeletal development. However, histological analyses of decalcified adult bone tissues revealed that the number and thickness of trabecular bones were reduced in *Cthrc1*-null mice, compared with their wild-type littermates (data not shown). This result suggested that Cthrc1 may regulate bone remodeling postnatally. To test this hypothesis, we performed bone histomorphometric analyses and microcomputed tomography (micro-CT) analyses of 2-month-old *Cthrc1*-null and wild-type mice. Micro-CT analyses of tibiae showed that the trabecular bone mass in *Cthrc1*-null mice was approximately 70% of that in wild-type mice (Figure 2 A and B). Bone histomorphometric and micro-CT analyses showed a significant decrease in trabecular number (Tb.N) and osteoblast number (Ob.N/BS) in *Cthrc1*-null mice, while trabecular thickness (Tb.Th) and tartrate-resistant acid phosphatase (TRAP)-positive osteoclast number (Oc.N/BS) had no change in *Cthrc1*-null mice (Figure 2C–E and Figure S4A). In addition, the osteoblast surface (Ob.S/BS) in *Cthrc1*-null mice, which represents the proportion of the bone surface covered with osteoblasts, was approximately 40% less than that in wild-type mice, and the osteoclast surface (Oc.S/BS), which represents the proportion of the bone surface covered with osteoclasts, showed no significant difference between *Cthrc1*-null and wild-type mice (Figure 2F and Figure S4A). To analyze osteoblast proliferation *in vivo*, we performed BrdU incorporation assays in the calvaria of *Cthrc1*-null and wild-type littermates, and found that the percentage of proliferating cells was decreased by approximately 30% in *Cthrc1*-null mice (Figure 2G). Moreover, double-labeling analyses with calcein, a marker of newly formed bone, showed that the bone formation rate was significantly decreased in *Cthrc1*-null mice (Figure 2H). Thus, these results indicate that the decreased bone mass in *Cthrc1*-null mice is due to the suppression of osteoblastic bone formation, not due to an acceleration of osteoclastic bone resorption.

### High bone mass and increased bone formation in transgenic mice overexpressing *Cthrc1* in osteoblasts

**Figure 3:**
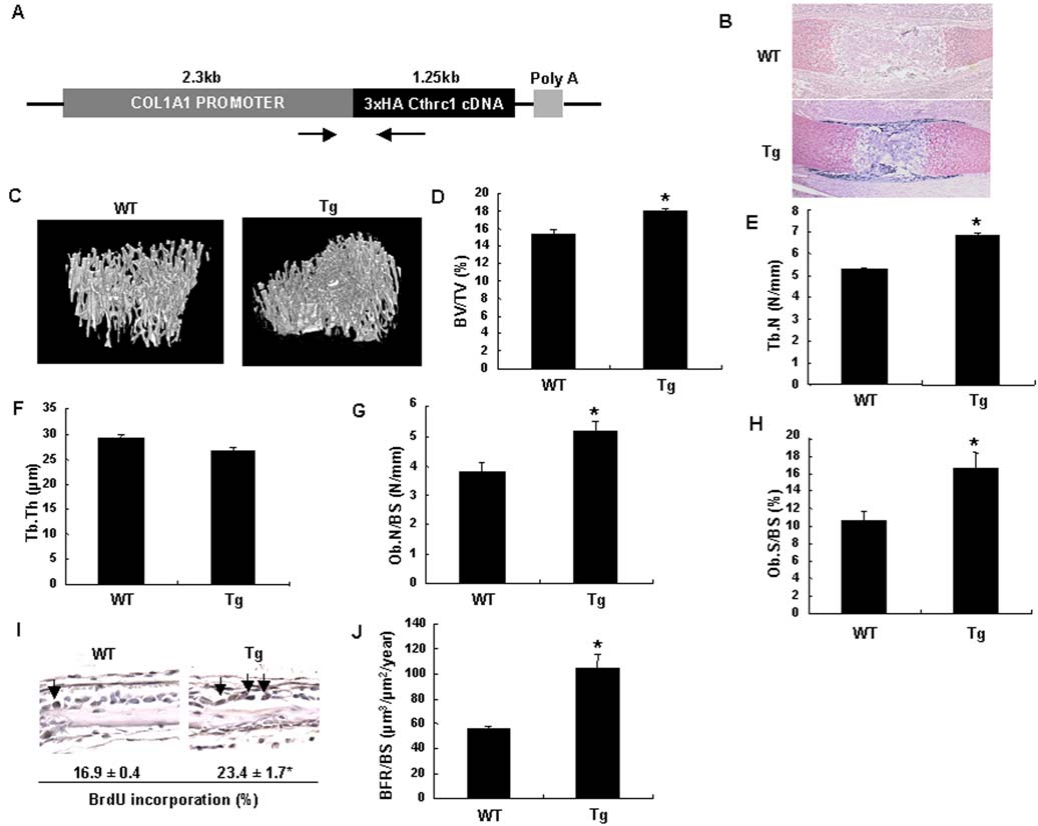
Generation of *Cthrc1* transgenic mice and analyses of bone phenotypes. (A) Schematic representation of the *Col1a1-Cthrc1* transgene. Poly A: SV40 polyadenylation signal. Arrows indicate the primers for genotyping. (B) Expression of 3×HA-tagged Cthrc1 protein in humeri of E16.5 *Cthrc1* transgenic mice. (C–F) Micro-CT analyses of tibiae in 2-month-old *Cthrc1* transgenic and wild-type mice (n = 6). Bone volume/total volume (BV/TV), trabecular number (Tb.N) and trabecular thickness (Tb.Th) are shown. (G and H) Bone histomorphometric analyses of vertebrae in 2-month-old *Cthrc1* transgenic and wild-type mice (n = 6). Osteoblast number/bone surface (Ob.N/BS) and osteoblast surface/bone surface (Ob.S/BS) are shown. (I) Analyses of osteoblast proliferation in calvaria of 1-week-old *Cthrc1* transgenic and wild-type mice by BrdU incorporation assays. Arrows indicate BrdU positive osteoblasts. (J) Calcein double-labeling of vertebrae in 2-month-old *Cthrc1* transgenic and wild-type mice. BFR: Bone formation rate. WT: wild-type mice; Tg: *Cthrc1* transgenic mice. Data are shown as the mean±SEM (^*^ *p*<0.05).

To determine if Cthrc1 is sufficient to increase bone mass, we next generated transgenic mice that specifically overexpressed 3×HA tagged *Cthrc1* in osteoblasts under the control of the *Col1a1* 2.3 kb osteoblast-specific promoter (Figure 3A). Immunohistochemistry with antibody against the HA-epitope tag showed specific expression of 3×HA tagged protein in bone collars, periosteum, and trabecular bones (Figure 3B). Hemizygous *Cthrc1* transgenic mice were born at the expected Mendelian ratio and grew normally in comparison with their wild-type littermates (data not shown). Skeletal preparations showed no apparent skeletal phenotypes in newborn hemizygous transgenic mice (Figure S2C). In addition, *in situ* hybridization analyses revealed that overexpression of Cthrc1 in osteoblasts did not affect the expression of the osteoblast markers, *Runx2* and *Col1a1*, and chondrocyte markers, *Col2a1* and *Col10a1*, in E16.5 mouse embryos (Figure S3B). As we found an osteopenic phenotype in adult *Cthrc1*-null mice, we analyzed bone in adult *Cthrc1* transgenic mice. Micro-CT analyses of tibiae showed that the trabecular bone mass in hemizygous *Cthrc1* transgenic mice was approximately 25% greater than that in wild-type mice (Figure 3C and D). Likewise, bone histomorphometric and micro-CT analyses showed significant increase in trabecular number (Tb.N) and osteoblast number (Ob.N/BS) in transgenic mice, while trabecular thickness (Tb.Th) and TRAP-positive osteoclast number (Oc.N/BS) had no change between transgenic mice and wild-type mice (Figure 3E–G and Figure S4C). The osteoblast surface (Ob.S/BS) in transgenic mice was approximately 40% greater than that in wild-type mice, while there was no difference in the osteoclast surface (Oc.S/BS) between transgenic mice and wild-type mice (Figure 3H and Figure S4C). BrdU incorporation assays showed that the percentage of proliferating osteoblasts was increased by approximately 35% in transgenic mice (Figure 3I). Furthermore, double-labeling analyses with calcein showed a marked increase in the bone formation rate in transgenic mice (Figure 3J). Thus, these data indicate that the increase in bone mass in *Cthrc1* transgenic mice is due to the stimulation of osteoblastic bone formation.

### Cthrc1 stimulates osteoblast proliferation and differentiation

**Figure 4:**
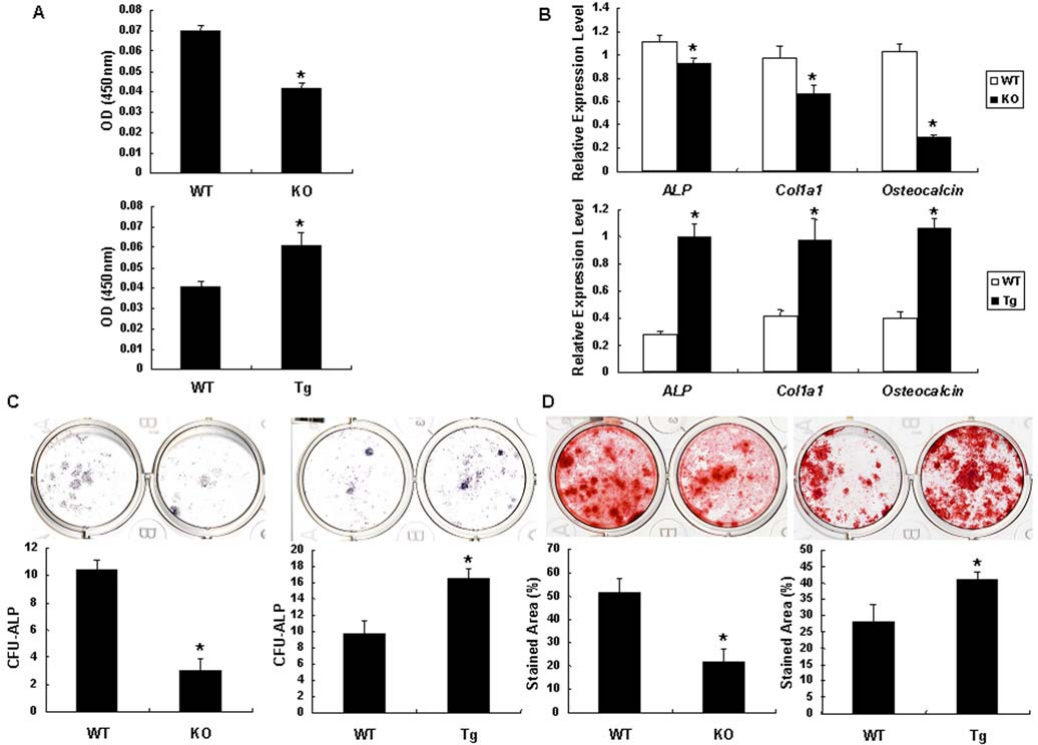
Effects of Cthrc1 on osteogenesis. (A) Cell proliferation in primary osteoblasts harvested from *Cthrc1*-null mice (upper panel) and from *Cthrc1* transgenic mice (lower panel) as shown by BrdU incorporation assays. (B) Expression of early and late osteoblast marker genes in primary osteoblasts harvested from *Cthrc1*-null mice (upper panel) and *Cthrc1* transgenic mice (lower panel). (C) The total numbers of CFU-ALP in bone marrow cell cultures derived from *Cthrc1*-null mice (left) and *Cthrc1* transgenic mice (right). (D) The mineralized area of CFU-O in bone marrow cell cultures derived from *Cthrc1*-null mice (left) and *Cthrc1* transgenic mice (right). WT: wild-type mice; KO: *Cthrc1*-null mice; Tg: *Cthrc1* transgenic mice. Data are shown as the mean±SEM (^*^ *p*<0.05).

Both the loss-of-function and gain-of-function analyses using mouse genetics approaches indicate that Cthrc1 positively regulates osteoblastic bone formation *in vivo*. To clarify the functions of Cthrc1 in osteoblasts, we isolated osteoblasts from the calvaria of wild-type (*Cthrc1* ^+/+^ osteoblasts) and *Cthrc1*-null (*Cthrc1* ^−/−^ osteoblasts) newborn mice and analyzed the effects of Cthrc1 on osteoblast proliferation and differentiation *in vitro*. BrdU incorporation in *Cthrc1* ^−/−^ osteoblasts was 40% less than that in *Cthrc1* ^+/+^ osteoblasts (Figure 4A). Real-time PCR analyses confirmed that the mRNA levels of the osteoblast marker genes, *ALP*, *Col1a1*, and *Osteocalcin* were all decreased in *Cthrc1* ^−/−^ osteoblasts, whereas the expression level of receptor activator of nuclear factor κB ligand (*RANKL*), a major determinant of osteoclastogenesis, did not differ (Figure 4B and Figure S4B). We further performed *in vitro* analyses of osteoprogenitor frequency and differentiation capacity by colony-forming unit (CFU) assays. CFU-ALP assays demonstrated that *Cthrc1* ^−/−^ bone marrow cells had dramatically reduced osteoprogenitors, and mineralized area of CFU-osteoblast (CFU-O) was also reduced by more than half in *Cthrc1* ^−/−^ bone marrow cells, consistent with decreased functional progenitors (Figure 4C and D). Likewise, we examined osteoblast proliferation and differentiation in osteoblasts derived from *Cthrc1* transgenic mice (Tg-osteoblasts) *in vitro*. As shown in Figure 4A, BrdU incorporation in Tg-osteoblasts was 25% greater than that in wild-type osteoblasts. The mRNA levels of *ALP*, *Col1a1*, and *Osteocalcin* were all increased in Tg-osteoblasts, whereas the expression level of *RANKL* did not differ (Figure 4B and Figure S4D). CFU assays showed that the frequency of osteoprogenitors and mineralized area of CFU-O were significantly increased in *Cthrc1* transgenic mice (Figure 4C and D). Thus, these results indicate that Cthrc1 accelerates bone formation by stimulating osteoblast proliferation and differentiation, resulting in increased postnatal bone volume.

### Cthrc1 attenuates ovariectomy (OVX)-induced bone loss

**Figure 5:**
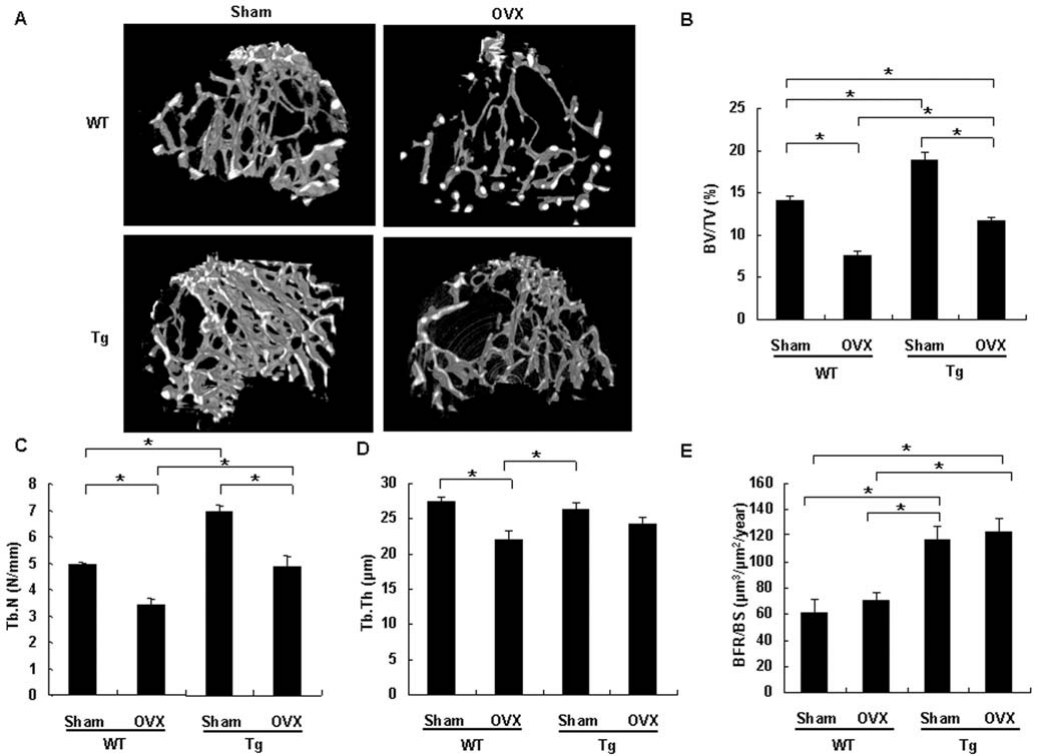
OVX-induced bone loss is attenuated in *Cthrc1* transgenic mice. (A) micro-CT of tibiae from 3-month-old *Cthrc1* transgenic and wild-type mice which were sham-operated or ovariectomized at 2 months of age (n = 5). (B–E) Bone volume/total volume (BV/TV) (B), trabecular number (Tb.N) (C), trabecular thickness (Tb.Th) (D) and bone formation rate (BFR) (E) were assessed by micro-CT or bone histomorphometry. WT: wild-type mice; Tg: *Cthrc1* transgenic mice. Data are shown as the mean±SEM (^*^ *p*<0.05).

In view of an anabolic effect of Cthrc1 on bone formation, we tested whether Cthrc1 may impact bone mass in the OVX mice, an established preclinical disease model of postmenopausal osteoporosis [16]. Eight-week-old *Cthrc1* transgenic and wild-type mice were ovariectomized or sham-operated, and four weeks after operation, metaphyseal regions of tibiae were analyzed by micro-CT. OVX induced 47% trabecular bone loss in wild-type mice, and 38%, in transgenic mice (Figure 5A and B), and trabecular bone mass in OVX transgenic mice was only 17% less than that in sham-operated wild-type mice. Trabecular number, which was reduced by ovariectomy in both wild-type and transgenic mice, did not significantly differ between sham-operated wild-type mice and OVX transgenic mice (Figure 5C). Trabecular thickness, which was reduced in OVX wild-type mice, had no significant difference between sham-operated and OVX transgenic mice (Figure 5D). These results indicated that stimulatory effect of Cthrc1 on bone formation partly prevents reduction in bone volume by ovariectomy. Indeed, increase in bone formation rates was maintained after ovariectomy in transgenic mice. (Figure 5E). Thus, Cthrc1 attenuates estrogen deficiency-induced bone loss by enhanced osteoclastic bone resorption through stimulating osteoblastic bone formation.

## Discussion

Bone mass is regulated by continuous remodeling which is based on the balanced action between osteoblastic bone formation and osteoclastic bone resorption [2]. This process is precisely coordinated to maintain the bone mass homeostasis mediated by multiple signaling pathways such as parathyroid hormone, transforming growth factor-β (TGF-β) and BMP [3], [7]. In this study, we show that Cthrc1 is involved in postnatal bone formation. In *Cthrc1*-null mice, bone volume, trabecular number, osteoblast number, and bone formation rates were significantly decreased. In *Cthrc1* transgenic mice, bone volume, trabecular number, osteoblast number and bone formation rates were increased. *In vitro*, CFU assays in bone marrow cells and analyses of expression of osteoblast specific genes, *ALP*, *Col1a1*, and *Osteocalcin*, in osteoblasts revealed that Cthrc1 stimulates differentiation of osteoprogenitor cells and osteoblasts. Furthermore, BrdU incorporation assays showed that Cthrc1 stimulates osteoblast proliferation *in vitro* and *in vivo*. In contrast, Cthrc1 did not affect either TRAP-positive osteoclast number and osteoclast surface *in vivo*, or *RANKL* expression levels in primary osteoblasts. These findings indicate that Cthrc1 acts as a stimulator of osteoblastic bone formation, but has no effect on osteoclastic bone resorption.

BMPs have potent osteogenic effects and control osteoblast proliferation and differentiation during osteogenesis [6], [12], [17]. Several studies of cell-specific inactivation or activation of BMP signals in mice have shown that BMPs regulate postnatal bone remodeling [8]–[12]. As shown in previous studies, Cthrc1 is regulated by TGF-β and BMP4, and a putative Smad binding site is identified in the promoter region of *Cthrc1* [13], [18]. We show that Cthrc1, which is induced by BMP2, is not required for skeletal development, but is for the maintainance of bone homeostasis, suggesting that Cthrc1 may act as one of the downstream targets of BMP-Smad signaling, followed by enhanced expression of osteoblast specific genes including *ALP*, *Col1a1 and Osteocalcin*.

The detail mechanism of Cthrc1 during bone formation is unclear. Cthrc1 inhibited TGF-β signaling in smooth muscle cells but not in endotherial cells [14]. Contrary to the effect of Cthrc1 on osteoblasts in *Col1a1* expression in our study, overexpression of Cthrc1 in PAC1 cells, smooth muscle cell line, reduced mRNA levels of *Col1a1*, and collagen deposition [13], [14]. These results suggest that the function of Cthrc1 differs among cell types, or that Cthrc1 signals through its own signaling pathway which is functional in a cell-type specific manner. In addition, there are discrepancies of phenotypes between *Cthrc1* transgenic mice which we generated in this study and the transgenic mice overexpressing *Cthrc1* previously reported [14]. First, transgenic mice we generated display a high-bone-mass phenotype, whereas transgenic mice previously reported show an osteopenic phenotype. Second, transgenic mice previously reported are smaller in size and have a higher rate of postnatal mortality, whereas transgenic mice we generated are fertile and normal in appearance and body weight. A possible explanation for these discrepancies is that transgenic mice we generated overexpress *Cthrc1* specifically in osteoblasts under the control of the *Col1a1* 2.3kb osteoblast-specific promoter to focus on the function of Cthrc1 in bones, while transgenic mice previously reported ubiquitously overexpress *Cthrc1* under the control of cytomegalovirus promoter.

Ovariectomy leads to a deficit in bone mineral densities due to cortical bone modeling and resorption of cancellous bone [19], [20]. This change largely results in severe cancellous osteopenia. Dempster et al. demonstrated that the primary mechanism of ovariectomy-induced bone loss is osteoclast perforation and removal of the trabecular plates [21]. Furthermore, a recent study showed that selective ablation of estrogen receptor α in differentiated osteoclasts induced trabecular bone loss, similar to the osteoporotic bone phenotype, caused by inhibition of osteoclast apoptosis [22]. Thus, ovariectomy-induced bone loss, a model of postmenopausal osteoporosis, results from an acceleration of osteoclastic bone resorption, followed by high bone turnover in which bone resorption exceeds bone formation. *Cthrc1*-null mice exhibit cancellous bone loss, and *Cthrc1* transgenic mice increase cancellous bone with no change in osteoclastic bone resorption. In addition, Cthrc1 stimulates osteoblast proliferation and differentiation and increases bone formation rates, suggesting that Cthrc1 prevents cancellous bone loss in ovariectomized mice by stimulating osteoblastic bone formation. Indeed, bone volume and trabecular number in OVX transgenic mice were higher than those in OVX wild-type mice, and the loss of trabecular thickness by ovariectomy was protected in transgenic mice. Moreover, increased bone formation rates in transgenic mice were maintained after OVX. Considering that Cthrc1 does not affect osteoclastogenesis, enhanced bone formation by overexpression of Cthrc1 may attenuate bone loss caused by enhanced osteoclastic bone resorption induced by OVX.

It is noteworthy in this article that Cthrc1 may have a potential role in treating osteoporosis, because we observed that osteoblast-specific overexpression of *Cthrc1* in mice attenuates bone loss which is induced by OVX, an accepted preclinical disease model of postmenopausal osteoporosis. The function of Cthrc1 as a stimulator of bone formation without affecting bone resorption is very promising, because most types of drugs which are currently used for the treatment of osteoporosis are antiresorptive in nature and insufficient for restoring bone volume in osteoporotic patients [23]. Although anabolic agents improve bone mass by stimulating osteoblast-mediated bone formation, only a single anabolic agent, parathyroid hormone 1–34 is available to treat osteoporosis at present [24], [25]. Herein, we indicate that Cthrc1 attenuates estrogen deficiency-induced bone loss by stimulating osteoblastic bone formation. Hence, Cthrc1 constitutes a potential target of an anabolic therapy for osteoporosis and the therapeutic effects are expected to be enhanced with antiresorptive drugs.

## Materials and Methods

### Suppression subtractive hybridization

ATDC5 cells were cultured for a total of 5 days in DMEM/Ham's F12 medium supplemented with 5% FBS and 10 µg/ml insulin with medium change every other day. Cells were exposed to 1 µg/ml BMP2 or vehicle for 10 hr. Poly (A)^+^ RNA was isolated from BMP2 -untreated and BMP2 -treated ATDC5 cells by a single-step method as previously described [26] and analyzed by suppression subtractive hybridization according to the manufacturer's instruction (PCR-Select cDNA Subtractions Kit, Clontech). After subtraction, the cDNAs were ligated into pCR2.1 (Invitrogen), and these subtracted cDNA libraries were further screened by differential hybridization (differential screening kit, Clontech). The cDNA fragment of approximately 500-bp expressed at a high level in BMP2 -treated ATDC5 cells was identified. Oligo (dT) primed cDNA library from poly(A)^+^ RNA of BMP2 -treated ATDC5 cells was constructed in λZAP Express vector (Stratagene), and 1×10^6^ plaques were screened with the 500-bp fragment as a probe as previously described [26].

### Generation of mutant mice

A Cthrc1 genomic clone was isolated from a mouse 129SvEv genomic DNA library. The targeting vector was constructed by inserting an IRES-LacZ-pA-loxP-flanked neomycin resistance expression cassette into exon 2 of the *Cthrc1* gene. Homologous recombination in 129SvEv ES clones harboring *Protamine 1-Cre* transgenes was identified by Southern blot analysis of SacI-digested genomic DNA using 5′ and 3′ probes located outside the homology regions used for gene recombination [27]. Mouse chimeras were generated by C57BL/6 host blastocyst injection of mutant embryonic stem cell clones, and the chimeras obtained were bred with C57BL/6 mice to generate heterozygous *Cthrc1* mice. Heterozygous mutants were then backcrossed eight times with C57BL/6 mice to generate mutant mice with a C57BL/6 genetic background. We then performed RT-PCR to determine the presence of *Cthrc1* transcripts. We used the following primers of *Cthrc1* for RT-PCR, based on exon3 and exon4: 5′-CTGCGAGTTCTGTTCAGTGG-3′ and 5′-GGGACTGAAATCGTCAGAGG-3′. *Cthrc1* transgenic mice were generated using an osteoblast-specific 2.3-kb *Col1a1* promoter, the 1.25-kb 3×HA-tagged full-length mouse *Cthrc1* cDNA, and the 240-bp SV40 polyadenylation signal [28]. DNAs were injected into pronuclei of fertilized C57BL/6×DBA/2 hybrid eggs, and the injected eggs were then transferred into CD1 foster mothers. Hemizygous mutants were backcrossed eight times with C57BL/6 mice to generate transgenic mice with a C57BL/6 genetic background. The tissue specificity of transgene expression was examined in immunohistochemistry studies. Routine mouse genotyping was performed by PCR. The following primer pairs were used: 5′-CATCAAGATGGTATAAAAGG-3′ and 5′-GCAGCAGCAGCACAAGGAAG-3′. The experimental protocols were approved by the Animal Care and Use Committee of Kyoto University.

### Histological analyses

Whole mount X-gal staining of embryos was performed as previously described [29]. For the histological analyses, we fixed embryos with 4% paraformaldehyde, embedded them in paraffin, and sectioned them into 7-µm-thick slices. Immunohistochemical staining was performed using peroxidase chromogens (Zymed)/TrueBlue substrate (KPL) with rabbit polyclonal anti-HA antibody (1∶500, Covance). For *in vivo* BrdU labeling, 1-week-old mice were injected intraperitoneally with 100 µg/kg BrdU (Amersham) and sacrificed 3 hr later. Sections were stained with anti-BrdU antibody (Amersham) and were counterstained with hematoxylin according to the manufacturer's instruction.

### Micro-CT Analyses

High-resolution micro-CT scanning (SMX-100CT; Shimazu) was performed to measure morphological indices of metaphyseal regions of tibia as previously described [30]. Metaphyseal regions were scanned 100 times with a slice increment of 8 µm. The most proximal slice was defined as the plane where the growth plate had just disappeared. Material properties were calculated using a commercial software package (VG Studio Max1.2: Visual Science) [30].

### Bone Histomorphometry

The mice were injected subcutaneously with calcein (20 mg/kg body weight; Sigma) 10 and 3 days before sacrifice. Fifth lumber vertebrae were fixed with 4% paraformaldehyde for 18 hr at 4°C. Undecalcified bones were embedded in methylmethacrylate, and 4-µm-thick sections were prepared for bone histomorphometric analyses of adult mice as previously described [31]. Sections were stained with 1% toluidine blue. Static and dynamic histomorphometric analyses were performed according to standard protocols using Histometry RT (SYSTEM-SUPPLY) [32], [33].

### Osteoblast Isolation and Culture

Primary osteoblasts were harvested from calvaria of newborn mice by sequential collagenase digestion (Roche) and were maintained in α-MEM containing 10% FBS. BrdU incorporation was measured using the cell proliferation ELISA Biotrack kit (Amersham).

### Real-Time PCR

Primary osteoblasts were cultured in α-MEM containing 10% FBS, 50 µg/ml ascorbic acid, and 10 nM β-glycerophosphate for 10 days, and total RNA was isolated from the cultured cells by RNeasy Mini Kit (Qiagen) according to the manufacturer's instruction. Two µg of total RNA was reverse transcribed to cDNA with the use of Transcriptor First Strand cDNA Synthesis Kit (Roche). Real-time PCR was performed using the LightCycler system with the FastStart DNA Master SYBR Green (Roche). The following primers were used: *G3pdh*, 5′-TGTCCGTCGTGGATCTGAC-3′ and 5′-CCTGCTTCACCACCTTCTTG-3′; *ALP*, 5′-ACTCAGGGCAATGAGGTCAC-3′ and 5′-CACCCGAGTGGTAGTCACAA -3′; *Col1a1*, 5′-CTCCTGGCAAGAATGGAGAT-3′ and 5′-AATCCACGAGCACCCTGA-3′; *Osteocalcin*, 5′-AGACTCCGGCGCTACCTT-3′ and 5′-CTCGTCACAAGCAGGGTTAAG-3′.

### Measurement of CFU-ALP and CFU-O

Bone marrow cells were isolated as previously described [26]. Bone marrow cells from 5-week-old mice were plated into 12-well plates at 2.5×10^6^ cells per well for CFU-ALP assays and at 5×10^6^ cells per well for CFU-O assays, and were cultured in α-MEM containing 10% FBS, 50 µg/ml ascorbic acid, and 10 nM β-glycerophosphate. For CFU-ALP assays, cultures were stained at day 10 with Sigma alkaline phosphatase kit (Sigma), and colonies with >20 cells were counted. Cultures were stained with 1% alizarin red S (Wako) at day 20 for the CFU-O assay of *Cthrc1*-null mice, and at day 16 for that of *Cthrc1* transgenic mice, respectively. The stained area was calculated using the freeware Image-J (NIH).

### Ovariectomy

Mice were ovariectomized or sham-operated at 2 months of age, and all of the mice were killed 4 weeks later and the tibiae and vertebrae were removed for analyses.

### Statistical analyses

Statistical analysis was performed by Student's t test to determine the significance between groups. Wild-type and transgenic OVX and sham-operated mice were analyzed by ANOVA followed by Fisher's protected least significant difference. Values were considered statistically significant at *p*<0.05.

More methods were shown in Supporting Information Text S1.

## Supporting Information

Figure S1 Analyses of Cthrc1 expression in vitro and in vivo by northern blot and in situ hybridization. (A) Effect of BMP2 (1 µg/ml) on Cthrc1 expression in ATDC5 cells. Cthrc1 expression is upregulated by BMP2. (B) Expression of Cthrc1 in various cell lines. (C) Expression of Cthrc1 in adult mouse tissues. (D) In situ hybridization analysis of Cthrc1 expression in limb buds of E13.5 and E16.5 mouse embryos. (E) Expression of Cthrc1 during embryogenesis.
(8.89 MB TIF)

Figure S2 Skeletal preparation of Cthrc1-null and Cthrc1 transgenic mice. (A) Whole-mount X-gal staining of heterozygous Cthrc1 embryos during embryogenesis. (B and C) Skeletons of newborn Cthrc1-null mice (B) and Cthrc1 transgenic mice (C) stained by alcian blue followed by alizarin red. WT: wild-type mice; KO: Cthrc1-null mice; Tg: Cthrc1 transgenic mice.
(5.91 MB TIF)

Figure S3 In situ hybridization analyses of osteoblast and chondrocyte marker genes in Cthrc1-null and Cthrc1 transgenic mouse embryos. Runx2, Col1a1, Col2a1 and Col10a1 expression in humeri of E16.5 embryos. (A) Cthrc1-null mouse embryos. (B) Cthrc1 transgenic mouse embryos. WT: wild-type mice; KO: Cthrc1-null mice; Tg: Cthrc1 transgenic mice.
(10.15 MB TIF)

Figure S4 Effect of Cthrc1 on osteoclastogenesis. (A) TRAP staining of vertebrae of 2-month-old Cthrc1-null and wild-type mice. TRAP-positive osteoclast number/bone surface (Oc.N/BS) and osteoclast surface/bone surface (Oc.S/BS) are shown (n = 6). (B) Expression of RANKL in primary osteoblasts harvested from Cthrc1-null mice, assessed by real-time PCR. (C) TRAP staining of vertebrae of 2-month-old Cthrc1 transgenic and wild-type mice. TRAP-positive osteoclast number/bone surface (Oc.N/BS) and osteoclast surface/bone surface (Oc.S/BS) are shown (n = 6). (D) Expression of RANKL in primary osteoblasts harvested from Cthrc1 transgenic mice, assessed by real-time PCR. WT: wild-type mice; KO: Cthrc1-null mice; Tg: Cthrc1 transgenic mice. Data are shown as the mean±SEM (*p<0.05).
(9.83 MB TIF)

Text S1 Supplementary Methods.
(0.05 MB DOC)
