# Supplementary figures and images for: Cthrc1 Is a Positive Regulator of Osteoblastic Bone Formation
Source: PLoS One. 2008 Sep 9;3(9):e3174. doi: 10.1371/journal.pone.0003174 (PMC2527134; doi:10.1371/journal.pone.0003174)

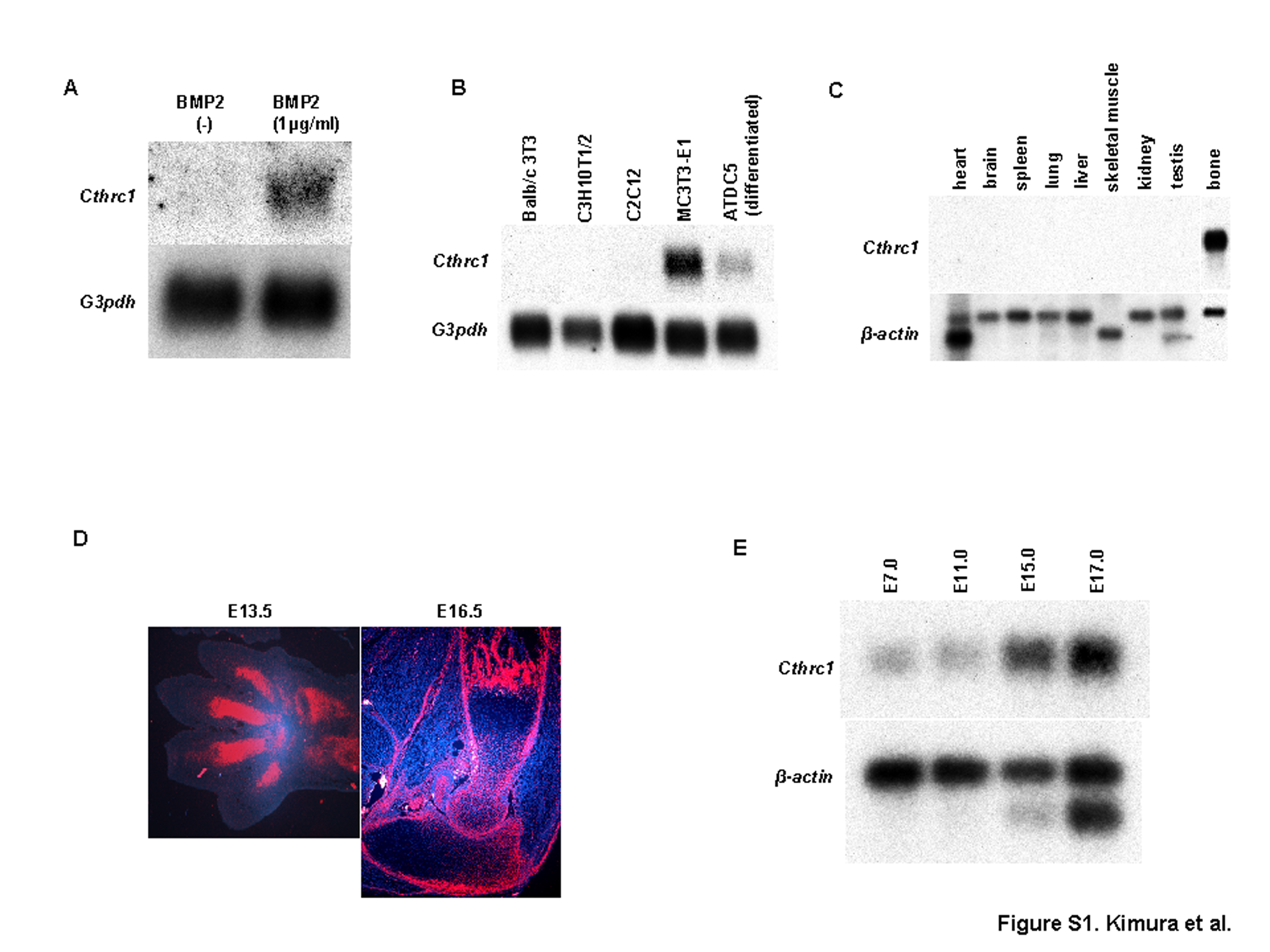

Supplement: Figure S1 — Analyses of Cthrc1 expression in vitro and in vivo by northern blot and in situ hybridization. (A) Effect of BMP2 (1 µg/ml) on Cthrc1 expression in ATDC5 cells. Cthrc1 expression is upregulated by BMP2. (B) Expression of Cthrc1 in various cell lines. (C) Expression of Cthrc1 in adult mouse tissues. (D) In situ hybridization analysis of Cthrc1 expression in limb buds of E13.5 and E16.5 mouse embryos. (E) Expression of Cthrc1 during embryogenesis. (8.89 MB TIF) [file pone.0003174.s001.tif]

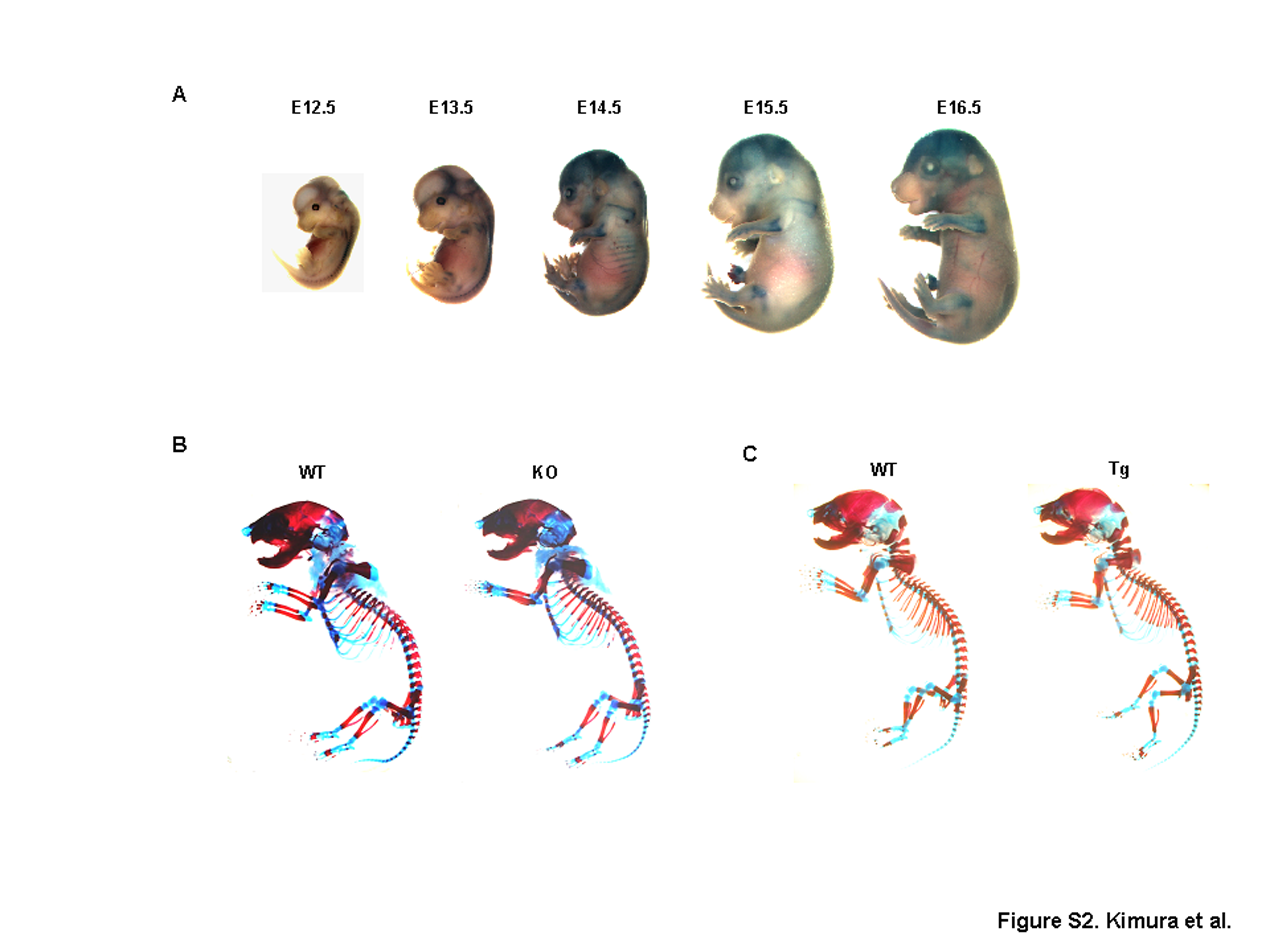

Supplement: Figure S2 — Skeletal preparation of Cthrc1-null and Cthrc1 transgenic mice. (A) Whole-mount X-gal staining of heterozygous Cthrc1 embryos during embryogenesis. (B and C) Skeletons of newborn Cthrc1-null mice (B) and Cthrc1 transgenic mice (C) stained by alcian blue followed by alizarin red. WT: wild-type mice; KO: Cthrc1-null mice; Tg: Cthrc1 transgenic mice. (5.91 MB TIF) [file pone.0003174.s002.tif]

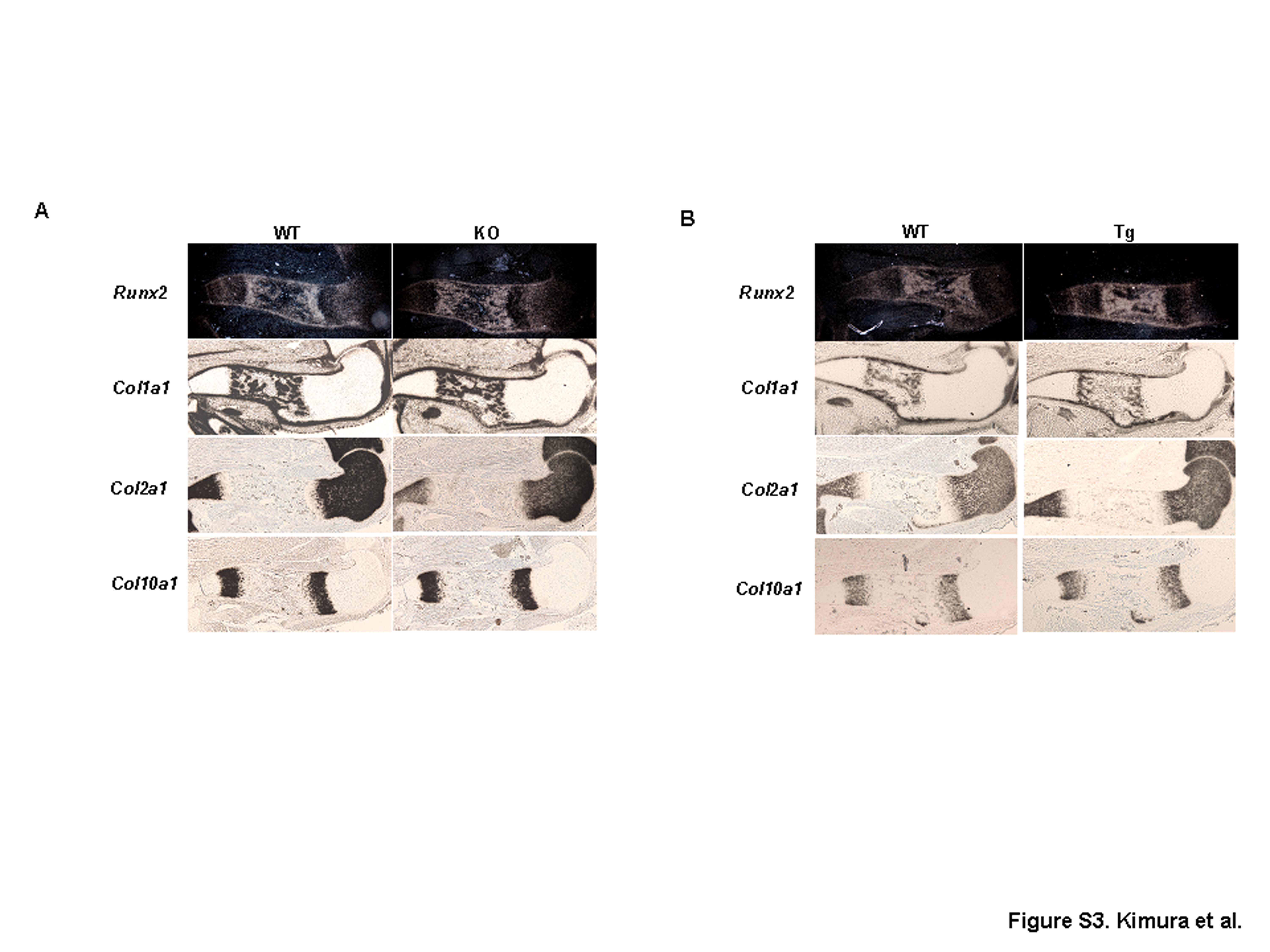

Supplement: Figure S3 — In situ hybridization analyses of osteoblast and chondrocyte marker genes in Cthrc1-null and Cthrc1 transgenic mouse embryos. Runx2, Col1a1, Col2a1 and Col10a1 expression in humeri of E16.5 embryos. (A) Cthrc1-null mouse embryos. (B) Cthrc1 transgenic mouse embryos. WT: wild-type mice; KO: Cthrc1-null mice; Tg: Cthrc1 transgenic mice. (10.15 MB TIF) [file pone.0003174.s003.tif]

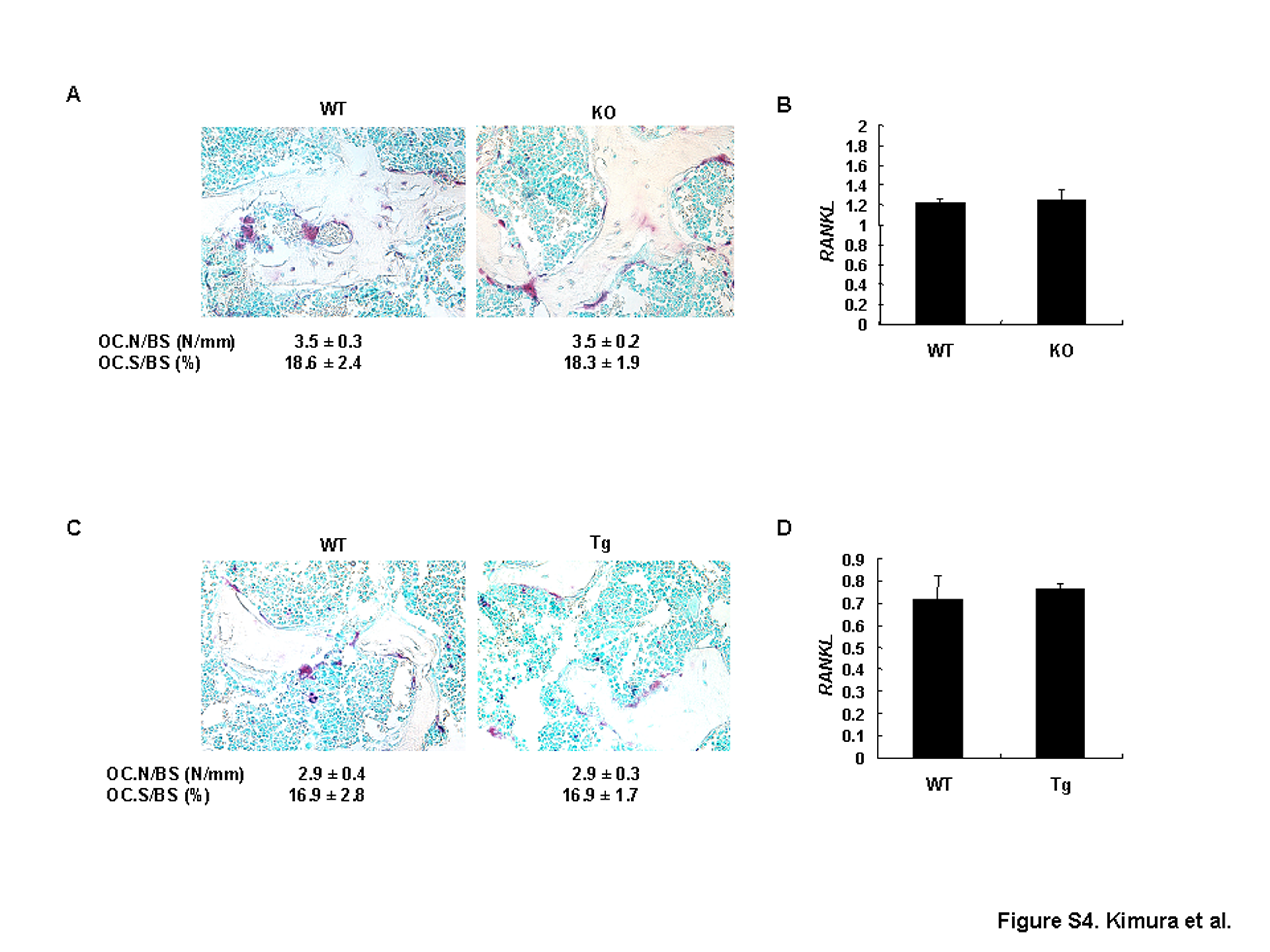

Supplement: Figure S4 — Effect of Cthrc1 on osteoclastogenesis. (A) TRAP staining of vertebrae of 2-month-old Cthrc1-null and wild-type mice. TRAP-positive osteoclast number/bone surface (Oc.N/BS) and osteoclast surface/bone surface (Oc.S/BS) are shown (n = 6). (B) Expression of RANKL in primary osteoblasts harvested from Cthrc1-null mice, assessed by real-time PCR. (C) TRAP staining of vertebrae of 2-month-old Cthrc1 transgenic and wild-type mice. TRAP-positive osteoclast number/bone surface (Oc.N/BS) and osteoclast surface/bone surface (Oc.S/BS) are shown (n = 6). (D) Expression of RANKL in primary osteoblasts harvested from Cthrc1 transgenic mice, assessed by real-time PCR. WT: wild-type mice; KO: Cthrc1-null mice; Tg: Cthrc1 transgenic mice. Data are shown as the mean±SEM (*p<0.05). (9.83 MB TIF) [file pone.0003174.s004.tif]
