# Supplementary material for: Cthrc1 Is a Positive Regulator of Osteoblastic Bone Formation
Source: PLoS One. 2008 Sep 9;3(9):e3174. doi: 10.1371/journal.pone.0003174 (PMC2527134; doi:10.1371/journal.pone.0003174)
Supplement: Text S1 — Supplementary Methods. (0.05 MB DOC) [file pone.0003174.s005.doc]

**Supporting information**

**Materials and methods.**

**Northern blot analyses**. Total RNA and poly (A)^+^ RNA from various cell lines were extracted, and northern blot hybridization was performed as previously described [1]. The following cDNA fragments were used as hybridization probes: a 500-bp fragment of *Cthrc1* cDNA and a 980-bp fragment of mouse glyceraldehyde 3-phosphate dehydrogenase (G3pdh) cDNA. In the analysis of *Cthrc1* expression in various tissues of adult mice, a labeled cDNA was hybridized to Mouse Multiple Tissue Northern (MTN) Blots (Clontech Laboratories, Inc.) and RNA extracted from mouse newborn calvaria.

***In situ* hybridization analyses.** Mouse embryos were fixed with 4% paraformaldehyde, dehydrated in a graded series of ethanol, and embedded in paraffin. Sections (7 µm thick) were then processed for *in situ* hybridization as previously described [2]. Subclones of cDNAs of marker genes into pBSII-KS(+) (Stratagene) was linealized with appropriate restriction enzymes to transcribe either sense or antisense [^35^S]-labeled riboprobes. After hybridization, the slides were washed under conditions of high stringency, and the dried tissue sections were dipped into NTB emulsion (Kodak, Rochester, NY) and exposed for 2-5 days at 4 °C.

**Skeletal preparation.** Whole-mount X-gal staining of embryos and alcian blue and alizarin red staining of the skeletons of newborn mice were performed as previously described [2,3]

**Real-Time PCR.** Primary osteoblasts were cultured in α-MEM containing 10% FBS, 50 μg /ml ascorbic acid, and 10 nM β-glycerophosphate for 10 days, and total RNA was isolated from the cultured cells by RNeasy Mini Kit (Qiagen) according to the manufacturer’s instructions. Two μg of total RNA was reverse transcribed to cDNA with the use of Transcriptor First Strand cDNA Synthesis Kit (Roche). Real-time PCR was performed using the LightCycler system with the FastStart DNA Master SYBR Green (Roche). The following primers were used: *G3pdh*, 5’-TGTCCGTCGTGGATCTGAC-3’ and 5’-CCTGCTTCACCACCTTCTTG-3’; *RANKL,* 5’- TGTACTTTCGAGCGCAGATG-3’ and 5’- CCCACAATGTGTTGCAGTTC-3’.

**Bone histomorphometry.** Undecalcified sections (4 μm thick) of fifth lumbar vertebrae were stained for tartrate-resistant acid phosphatase (TRAP) by using TRACP and ALP double-stain Kit (TaKaRa) and counterstained with methyl green to identify osteoclasts. Bone histomorphometric analyses were performed according to standard protocols using Histometry RT (SYSTEM-SUPPLY).

**References**

1. Akiyama H, Shigeno C, Hiraki Y, Shukunami C, Kohno H, et al. (1997) Cloning of a mouse smoothened cDNA and expression patterns of hedgehog signalling molecules during chondrogenesis and cartilage differentiation in clonal mouse EC cells, ATDC5. Biochem Biophys Res Commun 235: 142-147.

2. Akiyama H, Chaboissier MC, Martin JF, Schedl A, de Crombrugghe B (2002) The transcription factor Sox9 has essential roles in successive steps of the chondrocyte differentiation pathway and is required for expression of Sox5 and Sox6. Genes Dev 16: 2813-2828.

3. Nakashima K, Zhou X, Kunkel G, Zhang Z, Deng JM, et al. (2002) The novel zinc finger-containing transcription factor osterix is required for osteoblast differentiation and bone formation. Cell 108: 17-29.

**Legends for** **supporting figures**

**Figure S1.** **Analyses of *Cthrc1* expression *in vitro* and *in vivo* by northern blot and *in situ* hybridization.** (A) Effect of BMP2 (1μg/ml) on *Cthrc1* expression in undifferentiated ATDC5 cells. *Cthrc1* expression is upregulated by BMP2. (B) Expression of *Cthrc1* in various cell lines. (C) Expression of *Cthrc1* in adult mouse tissues. (D) *In situ* hybridization analysis of *Cthrc1* expression in limb buds of E13.5 and E16.5 mouse embryos. (E) Expression of *Cthrc1* during embryogenesis.

**Figure S2.** **Skeletal preparation of *Cthrc1*-null and *Cthrc1* transgenic mice.** (A) Whole-mount X-gal staining of heterozygous *Cthrc1* embryos during embryogenesis. (B and C) Skeletons of newborn *Cthrc1*-null mice (B) and *Cthrc1* transgenic mice (C) stained by alcian blue followed by alizarin red. WT: wild-type mice; KO: *Cthrc1*-null mice; Tg: *Cthrc1* transgenic mice.

**Figure S3. *In situ* hybridization analyses of osteoblast and chondrocyte marker genes in *Cthrc1*-null and *Cthrc1* transgenic mouse embryos.** *Runx2*, *Col1a1*, *Col2a1* and *Col10a1* expression in humeri of E16.5 embryos. (A) *Cthrc1*-null mouse embryos. (B) *Cthrc1* transgenic mouse embryos. WT: wild-type mice; KO: *Cthrc1*-null mice; Tg: *Cthrc1* transgenic mice.

**Figure S4. Effect of Cthrc1 on osteoclastogenesis.** (A) TRAP staining of vertebrae of 2-month-old *Cthrc1*-null and wild-type mice. TRAP-positive osteoclast number/bone surface (Oc.N/BS) and osteoclast surface/bone surface (Oc.S/BS) are shown (n=6). (B) Expression of *RANKL* in primary osteoblasts harvested from *Cthrc1*-null mice, assessed by real-time PCR. (C) TRAP staining of vertebrae of 2-month-old *Cthrc1* transgenic and wild-type mice. TRAP-positive osteoclast number/bone surface (Oc.N/BS) and osteoclast surface/bone surface (Oc.S/BS) are shown (n=6). (D) Expression of *RANKL* in primary osteoblasts harvested from *Cthrc1* transgenic mice, assessed by real-time PCR. WT: wild-type mice; KO: *Cthrc1*-null mice; Tg: *Cthrc1* transgenic mice. Data are shown as the mean ± SEM (^*^*p* < 0.05).
